# Genetic Interactions between the *Drosophila* Tumor Suppressor Gene *ept* and the *stat92E* Transcription Factor

**DOI:** 10.1371/journal.pone.0007083

**Published:** 2009-09-29

**Authors:** M. Melissa Gilbert, Carolyn K. Beam, Brian S. Robinson, Kenneth H. Moberg

**Affiliations:** Department of Cell Biology, Emory University School of Medicine, Atlanta, Georgia, United States of America; Institut Pasteur, France

## Abstract

**Background:**

Tumor Susceptibility Gene-101 (TSG101) promotes the endocytic degradation of transmembrane proteins and is implicated as a mutational target in cancer, yet the effect of TSG101 loss on cell proliferation in vertebrates is uncertain. By contrast, *Drosophila* epithelial tissues lacking the TSG101 ortholog *erupted* (*ept*) develop as enlarged undifferentiated tumors, indicating that the gene can have anti-growth properties in a simple metazoan. A full understanding of pathways deregulated by loss of *Drosophila ept* will aid in understanding potential links between mammalian TSG101 and growth control.

**Principal Findings:**

We have taken a genetic approach to the identification of pathways required for excess growth of *Drosophila* eye-antennal imaginal discs lacking *ept*. We find that this phenotype is very sensitive to the genetic dose of *stat92E*, the transcriptional effector of the Jak-Stat signaling pathway, and that this pathway undergoes strong activation in *ept* mutant cells. Genetic evidence indicates that *stat92E* contributes to cell cycle deregulation and excess cell size phenotypes that are observed among *ept* mutant cells. In addition, autonomous Stat92E hyper-activation is associated with altered tissue architecture in *ept* tumors and an effect on expression of the apical polarity determinant *crumbs*.

**Conclusions:**

These findings identify *ept* as a cell-autonomous inhibitor of the Jak-Stat pathway and suggest that excess Jak-Stat signaling makes a significant contribution to proliferative and tissue architectural phenotypes that occur in *ept* mutant tissues.

## Introduction

The *Drosophila* gene *erupted* (*ept*) encodes an ortholog of human Tumor Susceptibility Gene-101 (TSG101) and yeast Vps23p, which function as part of the ESCRT (endosomal sorting complex required for transport)-I complex to sort proteins to the multi-vesicular body for ultimate lysosomal degradation [1]–[3]. Mutations in the *ept/tsg101* gene (referred to hereafter as *ept*) or the ESCRT-II subunit gene *vps25* block endocytic degradation of certain transmembrane proteins and lead to dramatic overgrowth of imaginal discs [4]–[7], a set of polarized epithelial tissues that grow during larval stages and go on to form the majority of adult structures. This imaginal disc overgrowth occurs in part by a non-cell autonomous process: *ept* and *vps25* mutant cells themselves undergo very high rates of apoptosis, but can potently induce growth of surrounding genetically normal tissue due to overproduction of secreted factors like Unpaired (Upd) [4]–[6], a secreted cytokine-like mitogen that activates Jak-STAT signaling via the receptor Domeless (Dome) [8]. Because the death of *ept* and *vps25* mutant cells eventually limits Upd production, the phenotypic outcome of clonal loss of either gene is limited to enlargement of the affected organ. However, both *ept* and *vps25* also display a conditional, cell-autonomous tumor suppressor activity: if the death of mutant cells is prevented, either by expressing of anti-apoptotic genes or by removing competing normal cells from the disc, they overgrow into large, disorganized tumors that kill the host animal [4]–[6]. Thus *ept* and *vps25* behave both as non-cell autonomous growth suppressors, and as conditional, cell-autonomous neoplastic tumor suppressors.

The vertebrate *ept* gene homolog, TSG101, was first identified based on its ability to inhibit cell transformation [9] and by its apparent mutational inactivation in some cancers [10]. Later studies were unable to confirm key elements of these data [e.g. 11], [12]–[14], and subsequent analysis of mice carrying a targeted deletion of the TSG101 gene found no effect on cancer incidence or progression but did reveal a requirement for embryonic viability [15], [16]. More recent work showing that the TSG101 gene is overexpressed in some human cancers [17]–[19] and that transgenic overexpression of TSG101 in the mouse mammary gland mildly increases the frequency of breast carcinoma [17] tends to support the idea that excess TSG101 promotes, rather than inhibits, cell survival and proliferation. Since signaling via the EGF receptor, which is sorted to the lysosome via an ESCRT-dependent pathway in mammalian cells [20]–[23], is elevated in cells that overexpress TSG101 [17], excessively high levels of TSG101 may be capable of acting in a dominant-negative manner to reduce endocytic degradation of its normal targets thereby enhancing cell proliferation. A similar model has been proposed to explain a block in trafficking induced by overexpression of Vps18, a component of the mammalian Class C Vps/HOPS complex that interacts physically with TSG101 [24]. However, because genetic ablation of murine TSG101 does not produce hyper-proliferative phenotypes in vivo, the growth regulatory role of mammalian TSG101 remains uncertain.

The somewhat discordant views of the growth regulatory properties of TSG101-like proteins provided by mammalian and insect models suggest either that TSG101 is required for the endocytic degradation of a distinct set of receptors and membrane-associated proteins during vertebrate and invertebrate development, or that similar proteins enter the ESCRT pathway in each organism but other factors render *Drosophila* epithelia more susceptible to transformation by reduced ESCRT function. In order to lay the foundation for comparative analysis of vertebrate and invertebrate TSG101 developmental phenotypes, it is necessary to establish how *ept* mutations affect pathways that control cell proliferation and tissue growth. Here we show that the autonomous growth of *ept* mutant eye-antennal tumors is dependent on the *stat92E* gene, which encodes the sole fly member of the Signal Transducer and Activator of Transcription (STAT) family of mammalian transcription factors that are well known for their ability to promote tissue growth in *Drosophila* [25]–[27] and mammals [reviewed in 28]. Removing a single copy of *stat92E* gene significantly reduces the overgrowth of eye-imaginal discs composed entirely of *ept* cells. At a cellular level, this suppression by *stat92E* heterozygosity correlates with restoration of more normal G1/S cell cycle phasing in mutant discs and a partial reversion of an *ept* enlarged-cell phenotype. These effects on the proliferative properties of *ept* cells are accompanied by an unexpected ameliorating effect of *stat92E* heterozygosity on the epithelial architecture of *ept* mutant tissues. We find that lowering *stat92E* gene dosage reduces expression of the epithelial polarity factor Crumbs (Crb) in *ept* mutant eye discs. As excess Crb is sufficient to disrupt the architecture and polarity of epithelial tissues [29], [30], *stat92E* may thus contribute to *ept* tissue architectural phenotypes by affecting Crb expression (as we note above, an allele of *crb* failed to obviously alter the *ept* tumor phenotype; MMG and KHM, unpub.). To some degree the genetic evidence of a role for *stat92E* in *ept* phenotypes is not surprising: the excess Upd produced by *ept* mutant cells is known to drive Stat92E-dependent proliferation in immediately surrounding cells. However it has not been determined whether excess Stat activation contributes to the growth and polarity phenotypes of *ept* mutant cells themselves. We find that multiple sensors of Jak-Stat activity detect strong activation of the Jak-Stat pathway *within* mutant cells and that this phenomenon is coincident with accumulation of the Upd receptor Dome in intracellular puncta in *ept* mutant cells that co-stain with the endosomal protein Hrs [31]. Stat92E hyperactivity thus correlates with an autonomous effect of *ept* alleles on Dome localization and levels. In sum, these data indicate that *ept* is a cell-autonomous inhibitor of Jak-Stat signaling in the larval eye disc, and that elevated Stat activity contributes to deregulated cell cycling, excessive cell size, and defects in tissue organization observed in *ept* mutant tumors. These data provide a link between a TSG101-like protein and a pathway that controls cell proliferation and tissue architecture in a simple metazoan.

## Results

### Genetic requirement for *stat92E* in *ept* tumors

**Figure 1:**
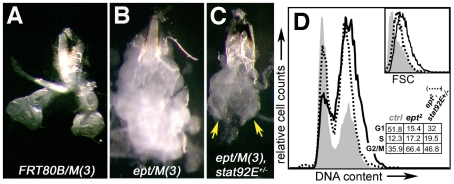
*stat92E* promotes growth of *ept* tumors. Bright-field images of (A) control discs [*FRT80B/M(3)*], (B) *ept* mutant discs [*ept^2^/M(3)*], or *ept* mutant, *stat92E* heterozygous discs [*ept^2^/M(3),stat92E^06346^/+*] from wandering-stage larvae. Yellow arrows in (C) denote the two lobes of tissue resembling eye discs. (D) Flow cytometric analysis of control (grey fill), *ept^2^/M(3)* mutant (black line), and *ept^2^/M(3),stat92E^06346^/+* (dotted line) eye-antennal discs shows that reducing *stat92E* gene dosage partially rescues of cell cycle and cell size (inset) defects in *ept^2^* mutant tissues. Percentages of cells in each stage of the cell cycle are indicated. The FACS data are representative of multiple experiments.

Eye-antennal discs were engineered to lack *ept* function by the *eyFLP,Minute* (*Min*) technique. In this configuration, mitotic recombination produces *Min/Min* cells that die and *ept* mutant cells that populate the disc and structures derived from it. These discs (hereafter referred to as ‘*ept/M(3)*’) overgrow into large unstructured tumors [5] (Fig. 1, compare A vs. B), indicating that loss of the gene can bypasses normal limits on organ size. To test the genetic requirements of this tumor-like phenotype, we screened a small collection of alleles of signaling, polarity, and growth regulatory genes (*stat92E*, *crb*, *lgl*, *Drosophila aPKC*, *yki, cyclinD*, *dMyc*, *s6k* and others) for their ability to suppress size and/or architectural phenotypes associated with loss of *ept/tsg101*. A single copy of the *stat92E^06346^* loss-of-function allele [32] was found to significantly reduce the size of *ept* eye-antennal tumors (Fig. 1C) and cause them to grow as two distinct tissue lobes (yellow arrows in Fig. 1C) rather than the large mass of tissue that otherwise characterizes *ept* mutant eye-antennal discs. This effect on organ size and architecture is completely penetrant, and is accompanied by a 15-fold increase in the frequency of pupation (0.4% of *ept/(3)* animals [n = 245] vs. 6% of *ept/M(3),stat92E^06346^/+* animals [n = 186]). To determine the effect of *stat92E* heterozygosity on the proliferative properties of cells within *ept* tumors, it was necessary to first establish the effect of *ept* loss on larval cell division and growth. Flow cytometric analysis indicates that cells within *ept* mutant tumors are enlarged relative to control cells (inset in Fig. 1D, black line vs grey fill) and show an increase in the percentage of cells in the S- and G2/M-phases of the cell cycle compared to cells in control *FRT80B/M(3)* discs (Fig. 1D, black line vs. grey fill). At a cellular level, the *stat92E^06346^* allele slightly reduces the size of cells within these tumors while increasing the fraction of G1 cells and decreasing the fraction of G2/M cells (Fig. 1D and inset; dotted line vs. black line). Thus a diploid dose of the *stat92E* gene is required for the effect of *ept* loss on G1/S cell cycle phasing and to a somewhat lesser degree for the enlarged size of *ept* mutant imaginal disc cells. As cells in *ept* tumors are highly proliferative [5], the former effect suggests that *stat92E* promotes S-phase entry in *ept* mutant cells. This conclusion agrees with published data showing that overexpression of the mitogen Upd in the eye disc increases the number of cells in S-phase [25].

### *stat92E* promotes overgrowth and architectural disorganization of *ept* tumors

**Figure 2:**
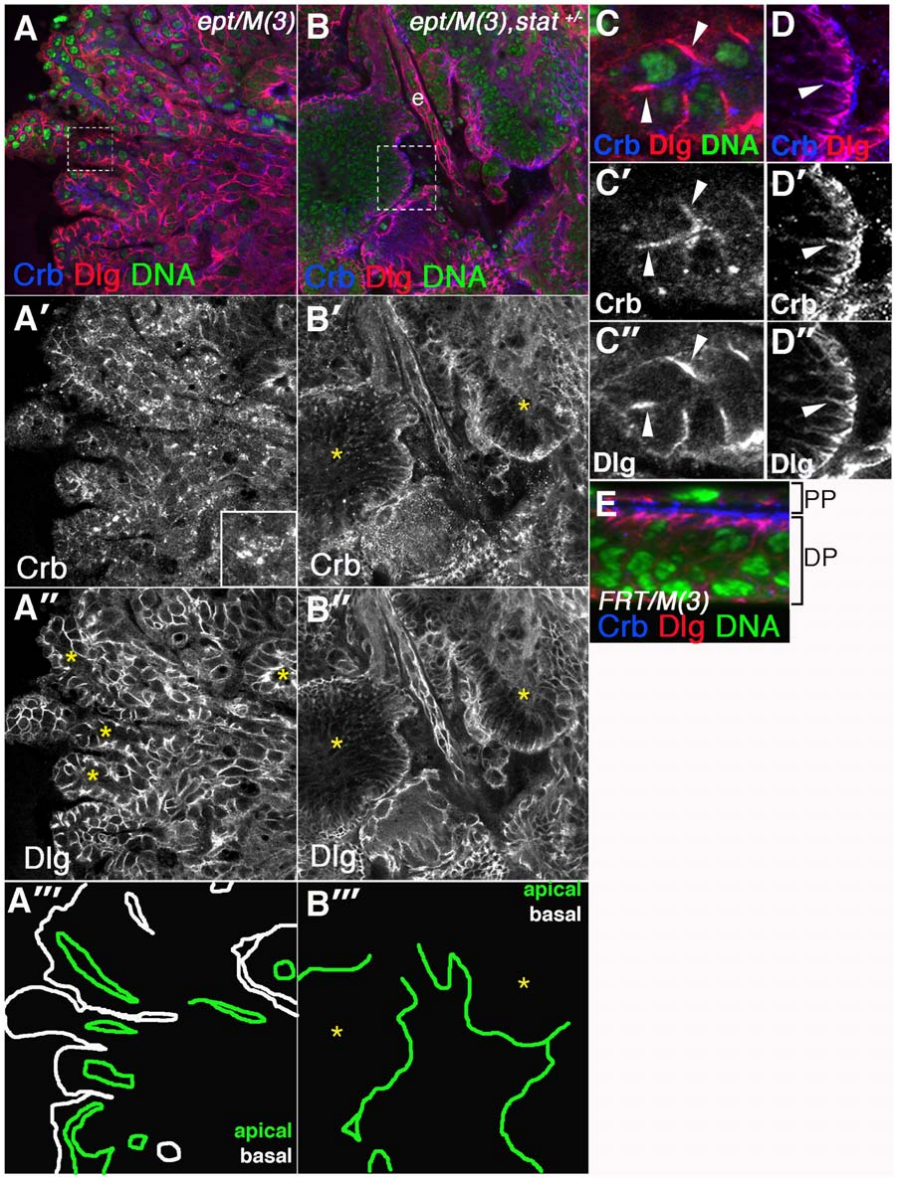
*stat92E* affects tissue architecture in *ept* tumors. Confocal images of *ept^2^/M(3)* (A-A″) and *ept^2^/M(3),stat92E^06346^/*+ (B-B″) eye discs stained for Dlg (red), Crb (blue) and DNA (green). The two main tissue lobes in panel B are separated by the esophagus (e), which remained embedded in the tissue mass during dissection. Areas outlined by dashed boxes in (A) and (B) are magnified in (C-C″) and (D-D″) respectively. Asterisks in (A″) denote internal lumens bounded by Dlg-negative apical membrane of *ept^2^/M(3)* mutant cells. Asterisks in B-B″′ denote tissue lobes of *ept^2^/M(3),stat92E^06346^/*+ discs. Note the dominant effect of *stat92E^06346^* on the appearance of Crb aggregates (A′ vs B′) and the lack of the ‘inverted’ tissue phenotype in *ept^2^/M(3),stat92E^06346^/*+ tumors (A″ vs B″). The *stat92E^06346^* allele does not have a dominant effect on the mislocalization of Crb onto the Dlg-positive basolateral membrane domain of *ept^2^* mutant cells (arrowhead in D-D″). (E) A control *FRT80B/M(3)* disc stained for Crb (blue), Dlg (red), and DNA (green). The peripodial cell layer (PP) and disc proper (DP) are indicated. Panels A″′ and B″′ are tracings of the apical (green) and basal (white) membranes of the tissues in A″ and B″ respectively.

Mutations in *ept* or *vps25* have previously been shown to alter epithelial polarity and tissue architecture [4]–[6]. The ability of the *stat92E^06346^* allele to restore a more normal morphology to *ept* mutant eye-antennal discs led us to examine the epithelial organization of these tissues. Co-staining for the basolateral membrane marker Discs large (Dlg) and the apical membrane determinant Crumbs (Crb) reveals that *ept* mutant eye-antennal discs are composed of compacted sheets of cells (Fig. 2A-A″) that form multilobular, ‘pouched’ structures with their apical surfaces oriented inward toward an internal lumen (examples denoted by yellow asterisks in Fig. 2A″). These structures are very much different from the normal monolayer organization of the peripodial and disc epithelium seen in control discs (Fig. 2E). Using the location of Dlg protein to trace the apical and basal surfaces of these *ept/M(3)* tissues highlights this phenotype (Fig. 2A′″). These architectural changes occur in parallel to a build-up of Crb protein in the cytoplasm of *ept* mutant cells (Fig. 2A′) due to a requirement for ESCRT complexes in vesicular trafficking of Crb [4], [5], [30]. Instead of concentrating at the apical surface of the tissue as in a control disc (Fig. 2E), Crb protein in *ept/M(3)* tumors is found more dispersed throughout the tissue (Fig. 2A′). A high magnification view of one of the ‘pouched’ structures (boxed in Fig. 2A) shows that *ept* is also required to prevent the spread of Crb from its normal location on the apical membrane onto the Dlg-positive basolateral domain (Fig. 2C-C″; arrowheads mark overlap of anti-Crb and anti-Dlg signals).

Tissue architecture was also analyzed in *ept* mutant discs also carrying a single copy of the *stat92E^06346^* allele. In this genotype, the multi-lobular ‘pouched’ structures do not form and the main masses of tissue (asterisks in Fig. 2B″) appear fairly solid with their apical, Crb-positive faces oriented outward (compare Fig. 2A″ to 2B″). Tracing the apical surface of these tissues emphasizes this architectural change (Fig. 2B″′). In addition, Crb aggregates are less abundant and Crb is more concentrated on the outer edge of the epithelium (compare Fig. 2A′ to B′). It is not clear whether these apparent effects on Crb levels and localization are independent, or whether a reduction in Crb levels leads to a less severe mislocalization phenotype. However, the membrane-associated pool of Crb in *ept,stat92E/+* mutant cells is still be detected overlapping the Dlg-positive basolateral domain (see arrowhead Fig. 2D-D″). Thus, a full genetic dose of the *stat92E* gene is required for the disruptive effect of *ept* loss on epithelial organization, and this appears to correlate with a requirement for *stat92E* in controlling Crb in *ept* mutant cells. By contrast, the mislocalization of Crb onto the basolateral membrane of *ept* mutant cells is either *stat92E*-independent or less sensitive to a two-fold reduction of *stat92E* dosage.

**Figure 3:**
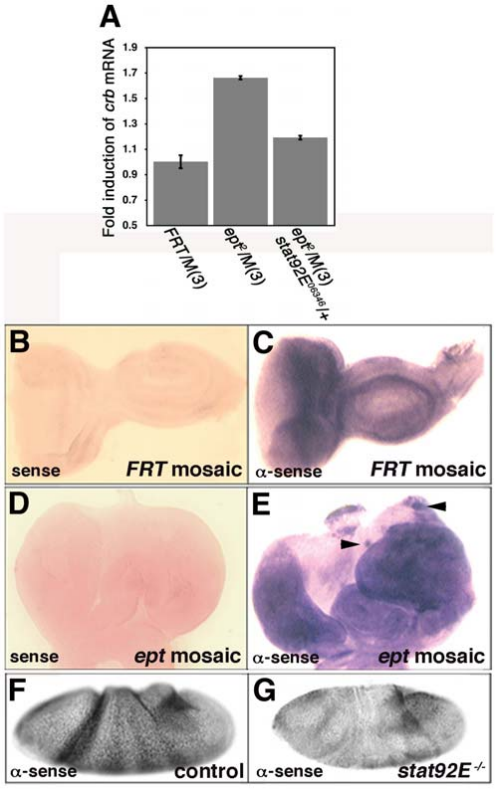
*stat92E* promotes *crb* expression in *ept* mutant cells. (A) *crb* transcript levels in the indicated genotypes as determined by quantitative realtime PCR. Levels of *crb* mRNA were normalized to control reactions to *rp49* mRNA. Data represents an average of two separate experiments. (B–G) RNA in situ hybridization analysis with *crb* sense (B,D) or anti-sense (C,E,F,G) probes in eye discs (B–E) or stage 3 embryos (F,G) of the indicated genotypes. Arrowheads in (E) indicate patches of cells that appear to contain higher levels of *crb* mRNA than surrounding cells.

In view of the effect of the *stat92E* genotype on anti-Crb staining phenotypes in *ept* eye-antennal tumors, we examined the relationship between *stat92E* gene dosage and *crb* mRNA levels. In situ hybridization with a *crb* specific probe detects ubiquitous *crb* expression in control mosaic eye discs (Fig. 3C) and in *ept* mosaic eye discs (Fig. 3D). In some cases, small patches of cells that appear to contain high levels of *crb* mRNA are detected in *ept* mosaic discs (arrowheads in Fig. 3D), although this effect is difficult to visualize against the high level of *crb* expressed in surrounding normal cells. Direct analysis of the level of *crb* transcript levels by quantitative realtime RT-PCR indicates that *crb* mRNA is induced ∼1.7-fold in *ept* mutant eye-antennal discs relative to control discs, and that this is suppressed by introduction of a single copy of the *stat92E^06346^* allele (Fig. 3A). Though these data indicate that *stat92E* is upstream of *crb* transcript levels in *ept* mutant cells, *stat92E* may not normally be required for *crb* expression in normal eye eye cells since Crb protein levels were unaffected in clones of eye disc cells homozygous for the *stat92E^06346^* allele (data not shown). However, *crb* transcript levels are markedly reduced in *stat92E^06346^* mutant embryos generated using the germ-line clone technique [33] (Fig. 3F–G). This link between *stat92E* and *crb* in the embryo could be due to indirect effects of *stat92E* loss on tissues that normally express *crb*. Yet the data linking *stat92E* and *crb* in *ept* tumors it is also quite consistent with the findings that *dome* function is required for Crb expression in ovarian follicle cells [34] and that Jak-Stat signaling is both necessary and sufficient for expression of *crb* in the posterior spiracles [35]. Loss of *ept* may thus affect Crb by two distinct mechanisms: first by a *stat92E*-dependent effect on *crb* mRNA levels, and second by defective endocytosis that prevents Crb protein degradation and sequesters it in the cytoplasm. Although reducing *crb* gene dosage in the background of *ept/M(3)* discs did not overtly affect tumor size or structure (data not shown), the role of Crb in the *ept* growth and tissue architectural phenotypes remains unclear.

### Activation of Jak-Stat signaling in *ept* mutant cells

**Figure 4:**
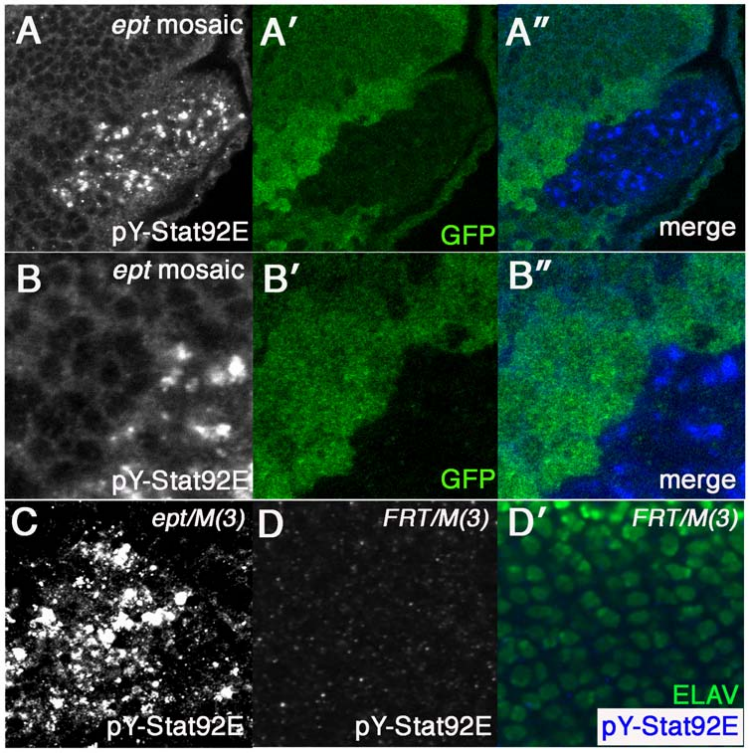
pY-Stat92E levels in *ept* mutant eye disc cells. A clone of *ept^2^* mutant eye disc cells (A–B) marked by the absence of GFP (green) and stains brightly for the pTyr-Stat92E epitope (blue). Note the pY-Stat92E epitope is specifically enriched within *ept^2^* mutant clones. Anti-pY-Stat92E signal in an *ept^2^/M(3)* eye-antennal tumor (C) and in a control *FRT80B/M(3)* eye antennal disc posterior to the morphogenetic furrow (D). Areas imaged in panels A, B, and D are posterior to the MF; the tumor in panel C has no MF.

**Figure 5:**
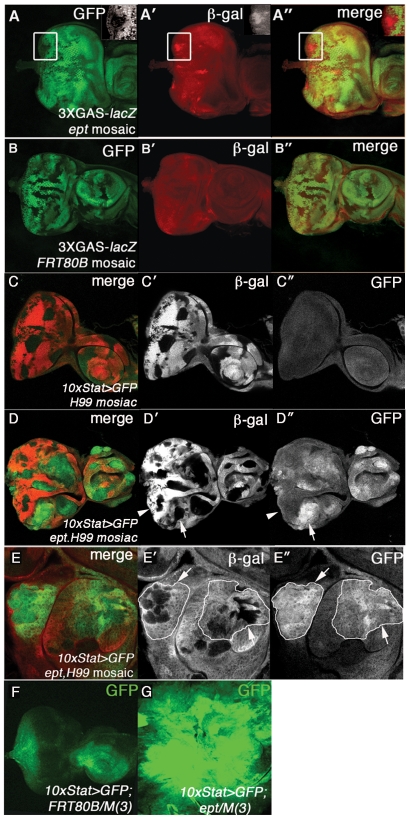
Stat92E sensor activity in *ept* mutant eye-antennal cells. Eye discs carrying clones of *ept^2^* mutant cells (A-A″) or *FRT80B* control cells (B-B″) marked by the absence of GFP (green) and stained for expression of β-galactosidase (β-gal; red in A′ and B′) to detect expression of the *3xGAS-lacZ* Stat-reporter. Inset in (A-A″) shows an *ept^2^* mutant clone that shows cell autonomous activation of *3xGAS-lacZ*. Because the *3xGAS-lacZ* transgene and the *ept* gene are both located on chromosome arm 3L, the reporter is present in two copies in both *FRT80B* and *ept* mutant clones; images in A′ and B′ were captured using exactly the same optical settings. (C–E) Expression of the *10xStat>GFP* transgene (green) in *ept^X1^,H99* mosaic discs in which mutant cells are marked by the absence of β-gal (red). Arrowhead marks clone of *ept^X1^,H99* mutant cells in the eye disc that do not activate *10xStat>GFP*; arrow marks an example of an antennal clone that activates *10xStat>GFP* within the clone and in surrounding wild type cells. Images in (E-E″) are of the antennal region of an *ept^X1^,H99* mosaic disc. White outlines in panel (E″) denotes boundaries of GFP-expressing cells. Expression of the Stat reporter *10xStat>GFP* (green) in a control *FRT80B/M(3)* eye disc (F) and an *ept^2^* eye-antennal tumor (G). The disc in (F) was imaged at half the fluorescence intensity relative to the control disc in (G).

Stat92E has been shown to be required for the non-autonomous pro-growth effect of *ept* mutant cells on surrounding normal cells [5]. The genetic requirement for *stat92E* in the overgrowth of *ept* mutant tumors indicates that the gene is also required for the autonomous growth effects of *ept* alleles. The mechanism of this effect is not known. *ept* mutant cells are known to overexpress the *upd* gene [5], which encodes a secreted cytokine-like ligand protein that binds the Dome receptor and signals through the Jak kinase to the Stat92E transcription factor [reviewed in 36]. As *upd* overexpression is alone sufficient to drive eye-antennal disc enlargement [25], high levels of extracellular Upd produced by *ept* cells might be predicted to feed back onto nearby cells (irrespective of their genotype) to elicit the growth and proliferation phenotypes observed in FACS analysis (see Fig. 1). To test the effect of *ept* alleles on Stat92E signaling, we first used an antibody reported to detect ligand-stimulated tyrosine phosphorylation of Stat92E by Jak kinase (anti-pY-Stat92E) which is necessary for Stat92E activity in vivo [reviewed in 37]. This antibody has been used in other studies of *Drosophila* Stat92E activity [7], [38], including one in which it was used to assess Jak-Stat activity in eyes discs mosaic for an allele of the ESCRT-II component *vps25* [7]. In eye-antennal discs composed of patches of normal and *ept* mutant cells, the anti-pY-Stat92E antibody detects strong accumulation of the pY-Stat92E epitope *within* clones of *ept* mutant cells (Fig. 4A-A″). A high magnification view of a clonal boundary confirms this effect (Fig. 4B-B″). This epitope is also elevated in mutant areas of *ept* disc tumors relative to wild type areas of *FRT80B/M(3)* control discs (Figs. 4C vs. D). To test the relationship between *ept* and Jak-Stat activity further, two Stat92E reporter transgenes were placed in the background of *ept* mutations. The first, *3xGAS-lacZ* [39], is located on the same chromosome arm as *ept*, such that mitotic recombination of a *3xGAS-lacZ,ept^2^,FRT80B* chromosome produces clones of *ept* cells carrying two copies of the *3xGAS-lacZ* transgene that can be compared to discs carrying control *FRT80B* clones with two copies of *3xGAS-lacZ*. When these cells are imaged under identical optical settings (Fig. 5A–B), the *3xGAS-lacZ* reporter is up-regulated in patches of *ept* eye disc cells (Fig. 5A-A″) relative to control *FRT80B* clones (Fig. 5B-B″). The inset in Fig. 5A shows a small *ept* clone in the posterior region of the eye disc that stains brightly for β-galactosidase expressed from the *3xGAS-lacZ* transgene. This small-clone phenotype is due to increased rates of apoptosis (Figure S1, and see [5]), which make it difficult to recover large *ept* mutant clones in the eye-antennal disc. To bypass this problem, expression of the second Stat92E reporter, *10xStat92E>GFP* [40], was analyzed in two backgrounds in which the death of *ept* mutant cells was blocked. The first of these was *ept/M(3)* tumors, in which removal of competing normal cells allows mutant cells to overgrow into large tumors [5]. *10xStat92E>GFP* is very strongly expressed in this background relative to its expression in control *FRT80B/M(3)* discs composed of normal cells (Fig. 5F–G). The magnitude of the difference is so substantial that the fluorescent signal from the disc in Fig. 5F was only detectable following a doubling of the ‘gain’ setting used to visualize the disc in Fig. 5G. *10xStat>GFP* was also analyzed in a second background in which the death of *ept* mutant cells was blocked by simultaneous loss of the *H99* chromosomal region, which contains genes required for apoptotic cell death [41]. We first confirmed that loss of the *H99* region alone had no effect on expression of *10xStat>GFP* (Fig. 5C). By contrast, expression of *10xStat>GFP* is very strongly up-regulated in eye-antennal discs carrying clones of *ept,H99* cells (Fig. 5D). An interesting pattern of GFP expression was observed in this experiment: some clones of *ept,H99* mutant cells located in the eye disc express *10xStat>GFP* highly both within clonal boundaries and in surrounding cells (see arrow in 5D′ and D″), whereas other nearby eye clones do not (see arrowhead in 5D′ and D″). This difference appears to be due to anterior/posterior positioning: clones that activate *10xStat>GFP* tend to be located anterior to the presumptive morphogenetic furrow, whereas those that do not tend to be located in posterior regions of the disc. Autonomous and non-autonomous activation of *10xStat>GFP* by *ept,H99* clones is also observed in the antennal disc (Fig. 5D–E). *10xStat>GFP* can be strongly activated both within clones and in cells located 5–10 cell diameters away from the mutant clones (Fig. 5E-E″, arrows and white outlines). This evidence of non-autonomous *10xStat>GFP* expression fits very well with previous models in which excess Upd produced by *ept* or *vps25* mutant cells is able to activate Stat in surrounding cells [5]. The more novel observation of autonomous *10xStat>GFP* activation within *ept,H99* mutant clones agrees with the data gathered with the pY-Stat92E and *3xGAS-lacZ* reporters. The differences in the readouts provided by these three pathway reporters also suggest that *10xStat>GFP* may be a more faithful reporter of Stat92E activity in larval discs than either pY-Stat92E or *3xGAS-lacZ*. In aggregate, these observations indicate that the effect of *stat92E* heterozygosity on *ept* tumor growth is a reflection of a role for Jak-Stat hyperactivity in the growth, cell cycle, and polarity characteristics of *ept* mutant cells.

### Cell-autonomous effect of *ept* loss on Dome

**Figure 6:**
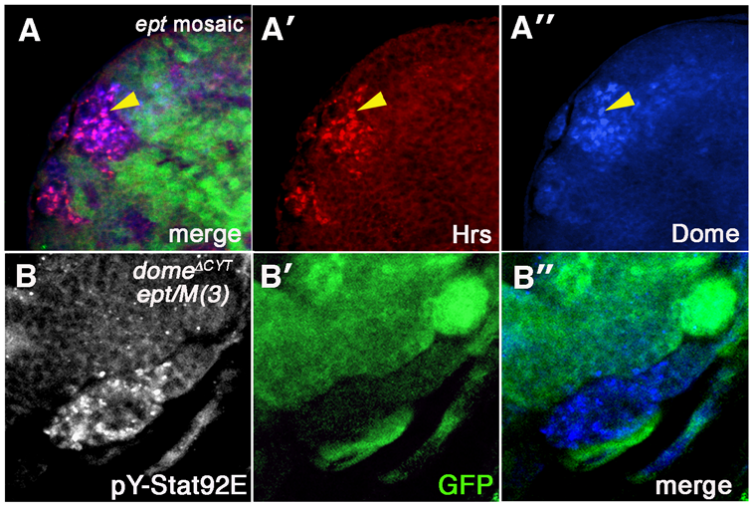
Dome localization in *ept* mutant tissue. (A-A″) A clone of *ept^2^* mutant eye disc cells marked by the absence of GFP (green) stained for Dome (blue) and the endocytic marker Hrs (red) shows extensive accumulation of Dome in Hrs-positive structures (yellow arrowhead denotes example of magenta overlap). (B-B″) A confocal image of a section of an *ept^2^* eye-antennal tumor expressing *dome^ΔCYT^* (using the *eyFLP;Actin>CD2>Gal4, UAS-GFP* system) and stained for anti-pYStat92E (blue); GFP (green) marks cells that express the *dome^ΔCYT^* transgene. The anti-pY-Stat92E epitope is strongly reduced in cells that express *dome^ΔCYT^* but not in the patch of GFP-negative, *ept^2^* mutant cells that do not express *dome^ΔCYT^*.

The autonomous effect of *ept* loss of pY-Stat92E suggests that this phenotype is not only an indication of Jak-Stat activation, but may also reveal a requirement for *ept* in controlling an intracellular step in the Jak-Stat cascade. Consequently, we sought to test whether *ept* loss effects trafficking of the transmembrane receptor Dome. Dome traffics through the late endosome [34], and loss of the *hrs* gene, which acts at the step immediately preceding *ept* [reviewed in 42], blocks Dome trafficking and activation even in the presence of Upd [43]. These observations have led to the proposal that movement of Dome into and through the endosomal system is a significant regulatory step in Jak-Stat signaling [43]. Since loss of *ept* leads to accumulation of certain apical trans-membrane proteins in the late endosome [5], we tested whether *ept* loss might also affect levels or localization of Dome. An antibody specific to the Dome protein [34] detects much higher levels of Dome in *ept* cells than in surrounding normal cells (Fig. 6A-A″). This Dome appears as puncta that partially co-localize with the endosomal protein Hrs (see arrowheads in Fig. 6). Loss of *ept* may also have more mild non-autonomous effects on Dome (see cells to the right of the clone in Fig. 5A″). Since Hrs-dependent movement of Dome into the late-endosome has been proposed to be required for activation of Stat92E [43], we next tested whether the accumulation of the pY-Stat92E epitope in *ept* mutant cells was dependent on Dome activity. The *Actin>CD2>Gal4* “flip-out” chromosome [44] was used in combination with a *UAS-GFP* transgene and a transgene carrying a dominant-negative form of *dome* that lacks the C-terminal tail (*UAS-dome^ΔCYT^*) [45] to produce GFP-positive/*dome^ΔCYT^*-expressing clones in the background of an *ept* tumor. *ept* mutant cells that express the *dome^ΔCYT^* allele (GFP-positive area in Fig. 6B-B″) do not display excess anti-pY-Stat92E staining, whereas those that do not express *dome^ΔCYT^* (GFP-negative area in Fig. 6B-B″) retain high levels of the pY-Stat92E epitope. From these data, we conclude that *ept* loss alters Dome localization and levels in eye imaginal disc cells and this correlates with Dome-dependent accumulation of the pY-Stat92E epitope. This data agrees with a proposed model in which Dome must access the Hrs-positive late endosome in order to activate signaling [43]. The trapping of Dome in this ‘activation’ compartment in *ept* mutants may therefore contribute to high-level activation of the Jak-Stat pathway observed in these cells.

## Discussion

**Figure 7:**
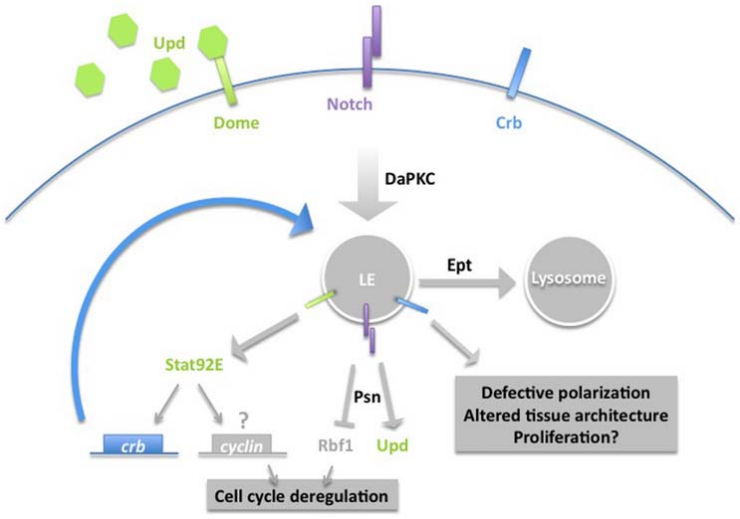
A hypothetical model of the cell-autonomous effects of *ept* loss. This model incorporates data from this paper and an accompanying study on the link between *ept*, DaPKC, Notch, Crb, Psn and Rbf1. The Erupted protein (Ept) is required to traffic Crb (blue), Notch (purple), and Dome (green) into the lysosome. DaPKC may regulate an upstream step in this process by promoting internalization of Notch and Crb. Loss of Ept causes Notch, Crb, and Dome (and perhaps other unidentified receptors) to accumulate in the endosome, from which they drive downstream effects. Stat92E likely has many nuclear targets in *ept* mutant cells, including *crb*. Given the links between mammalian Stats and G1 cyclin expression, Stat92E may have the potential to affect expression of a cyclin as well. Presenilin (Psn) acts upstream of Rbf1, perhaps via its role in activating Notch; a similar role for Psn upstream of the Notch-target Upd is untested but would follow logically from the available data. The excess Crb expressed in *ept* mutant cells is predicted to cycle back into the late endosome (LE), leading to very high levels of vesicular Crb. Overexpressed Crb is known to disrupt tissue architecture (e.g. [30]) and may to do the same in the context of *ept* tumors.

We have sought to identify pathways that mediate the cell-autonomous growth suppressor activity of the *Drosophila* endocytic gene *ept*, which encodes a homolog of mammalian Tumor Susceptibility Gene-101. We find that *ept* is required in vivo to restrict cell-autonomous activation of the Jak-Stat pathway, a well-established oncogenic pathway in mammals. This correlates with effects of the Drosophila *stat92E* gene on G1/S cell cycle control, cell size, and epithelial organization of *ept* mutant tumors. We also find that trafficking of Dome, which acts upstream of Stat92E, is altered in *ept* mutant cells. A previous study in the cultured *Drosophila* hemocyte S2 cell line also identified *ept* as a negative regulator of Jak-Stat signaling [46], but the extent to which this relationship is conserved in developing tissues in the whole organism and its contribution to *ept* loss-of-function phenotypes were not addressed. The data presented here suggest that excess Stat92E activity contributes to deregulation of the G1-to-S phase cell cycle transition and promotes growth of *ept* mutant cells and tissues. We also find evidence that *stat92E* promotes accumulation of the *crb* mRNA in *ept* mutant cells, and that this correlates with an effect of *stat92E* gene-dosage on Crb and epithelial architecture in *ept* mutant tumors. These findings, and those of an accompanying study, are summarized in Figure 7.

Since Upd can stimulate the endocytic uptake of Dome [34], the effect of *ept* loss on Dome protein could theoretically be a secondary consequence of the fact that these cells express highly elevated levels of Upd. In addition, it has been shown that *dome* itself is a transcriptional target of Stat92E as part of a positive feedback loop [25]. Alternatively, the relationship between *ept* and Dome localization could indicate a direct requirement for *ept* in Dome endolysosomal trafficking in much the same way that ESCRT mutants block the vesicular movement and lysosomal turnover of the Notch receptor [4]–[6], [47]. The simple observation that Dome can be trapped in an Hrs-positive compartment agrees with other studies that have shown that the Dome receptor also fluxes through the ESCRT endosomal system in imaginal disc and ovarian follicle cells, and that endocytic trafficking of Dome can modulate the output of the downstream Jak-Stat pathway [34], [43]. The oncogenic properties of this pathway are well-established in flies [25], [27], [48]–[50] and mammals [reviewed in 28], but its pro-growth targets are not fully understood. The data presented here support a role for Stat92E in promoting cell division and to a lesser degree cell size in *ept* mutant eye disc cells. A genetic screen for suppressors of a large-eye phenotype produced by misexpression of *upd* identified multiple components of the *dpp* (TGFβ) pathway [25], suggesting that Jak-Stat promotes growth via *dpp* signaling. However in the same study, activation of the *dpp* pathway was insufficient to rescue the small eye size of *upd* mutants, suggesting that additional pathways are responsible for Upd-dependent organ growth. One of these may involve Cyclin D, the cell cycle regulatory molecule that can promote tissue growth in *Drosophila* [51], [52] and cancer in mammals [reviewed in 53]. Mammalian STAT5 is known to bind and transactivate the CyclinD1 promoter [54], and more recently misexpression of upd has been found to induce *cyclin D* in the Drosophila eye [27]. These findings offer a potentially more direct link between Jak-Stat and cell proliferation control in imaginal disc cells and provide a basis for further studies of growth pathways activated in *ept* tumors.

The *Drosophila* and mammalian forms of TSG101 are quite similar at a primary sequence level (46% identical/61% similar), share the same domain structure [5], and are predicted to have very similar molecular properties. Each has also been shown to function as part of the same conserved complex, ESCRT-I, and to be involved in the same biological process: endocytic trafficking of internalized receptors and membrane proteins. Thus the observed differences in the phenotypes elicited by loss of vertebrate and invertebrate TSG101 are likely to arise either due to 1) differences in the strength of alleles used in each system, 2) differences in the spectrum of proteins routed into the ESCRT pathway in flies and mammals, or 3) functional redundancy for TSG101 within the vertebrate genome. With regard to the former possibility, the genomic alleles used for analysis in both organisms are loss of function embryonic lethal [5], [16], [55] that can be rescued by reintroduction of a wild-type version of the gene [5], [15]. In addition, TSG101 does not appear to be a member of a multi-gene family in vertebrates. Differences in cellular phenotypes produced by invertebrate and vertebrate *ept/TSG101* alleles thus may reflect stage- or tissue-specific differences in the spectrum of proteins routed into the ESCRT pathway in each type of organism. In addition to Crb and Notch, *Drosophila ept* has now been shown to affect localization and levels of the Dome receptor (see model in Fig. 7). Loss of the ESCRT-II subunit and tumor suppressor gene *vps25* additionally affects trafficking of the Tkv type-1 TGFβ receptor [6], and loss of the *hrs* gene affects a wide spectrum of cell surface receptors [56]. It remains to be determined whether or not the effect of *ept* on Dome is direct, and whether Dome homologs in other species are also affected by alterations in ESCRT-mediated trafficking. However if these proteins require TSG101 to traffic through the ESCRT pathway in a specific subset of mammalian epithelia, it may be that loss of TSG101 function in these tissues will result in growth phenotypes similar to those observed in *Drosophila* imaginal disc epithelia lacking *ept*.

## Materials and Methods

### Genetics

Crosses were done at 25°C unless otherwise indicated. *ept* clones were generated by crossing *w;ept^2^,FRT80B/TM6B* and *yweyFLP;P[m-w^+^;ubiGFP]*,*FRT80B*. *ept,H99* clones were generated by crossing *w;ept^X1^,H99,FRT80B/TM6B* and *yweyFLP;P[m-w^+^;ubiGFP]*,*FRT80B*. *ept* mutant eye-antennal tumors were generated by crossing *w;ept^2^,FRT80B* and *yweyFLP;P[m-w^+^]RpL14^1^,FRT80B/TM6B*. The *3xGAS-lacZ* reporter was placed into control or *ept* mutant backgrounds by crossing *3xGAS-lacZ,FRT80B/TM6B* or *3xGAS-lacZ,ept^2^,FRT80B/TM6B* males to *yweyFLP;P[mw^+^;ubiGFP]*,*FRT80B* females. *10xStat-GFP* (gift of E. Bach) activity was measured by crossing *10xStat-GFP;ept^2^FRT80B/TM6B* or *10xStat-GFP;FRT80B* to *yweyFLP;P[m-w^+^]L14^1^,FRT80B/TM6B*. ‘*ept,stat92E^06346^/+*’ animals were obtained by crossing *w;ept^2^,FRT80B,stat92E^06346^/TM6B* to *yweyFLP;P[w^+^]RpL14^1^,FRT80B/TM6B*. ‘*DN-dome; ept/M(3)*’ animals were obtained by crossing *UAS-dome^ΔCYT^*;*ept/TM6B* and *yweyFLP;act<y^+^<Gal4/*CyO:*twi-*GFP;*P[m-w^+^]RpL14^1^ FRT80B/TM6B* animals.

### Molecular biology

Total RNA from *FRT80B/M(3), ept/M(3)* and *ept/M(3), stat92E^06346^/+* animals was isolated using TRIzol (Invitrogen) and reverse transcribed (SuperScript II RT/Invitrogen). *crb* transcript was analyzed by qPCR (SYBR Green 1 Master/Roche). *rp49* was used as a control to normalize *crb* transcript levels in each sample. Primers used: *crb* 5′-cgtgctcgtttgacagttgta-3′ and 5′-cgattcggagtgcgtagg-3′; *rp49* 5′-cttcatccgccaccagtc-3′ and 5′-cgacgcactctgttgtcg-3′. RNA in situ hybridization was performed as described previously [57]. A DIG labeled *crb* riboprobe was synthesized from the linearized *crb* cDNA (DGRC) and visualized with anti-DIG-AP (1∶2000, Roche).

### Flow Cytometry

Discs were dissociated in PBS Trypsin-EDTA, 20 µM DRAQ-5 (Biostatus Limited). Sample data were acquired on a Becton Dickinson LSR II flow cytometer via a 755 nM Red laser with a 780/60 nM BP collection filter and analyzed with FACSDiva Software.

### Microscopy & Immunohistochemistry

Immunostaining and confocal microscopy was performed as described previously [58]. Antibodies used: rat anti-Crb-extra (gift of U. Tepass and E. Knust) 1∶500; guinea pig anti-Hrs (gift of H. Bellen) 1∶1000; rabbit anti-pYStat92E (Cell Signaling) 1∶1000; mouse anti-Dlg (DSHB) 1∶20; rabbit anti-Domeless-intra (gift of S. Noselli) 1∶200; goat anti-rabbit Cy5, goat anti-mouse Cy3, goat anti-guinea pig Cy3, and goat anti-rat Cy3 (Jackson Laboratories) each at 1∶50; YOYO (Molecular Probes) was used at 1∶2000.

## Supporting Information

Figure S1 Rates of death in *ept/tsg101* mutant eye-antennal clones. A clone of *ept/tsg101* mutant cells in the eye disc marked by the absence of GFP (green) stain brightly for the cleaved, activated form of Caspase-3 (blue).
(0.54 MB TIF)
