# Supplementary figures and images for: Genetic Interactions between the Drosophila Tumor Suppressor Gene ept and the stat92E Transcription Factor
Source: PLoS One. 2009 Sep 29;4(9):e7083. doi: 10.1371/journal.pone.0007083 (PMC2747001; doi:10.1371/journal.pone.0007083)

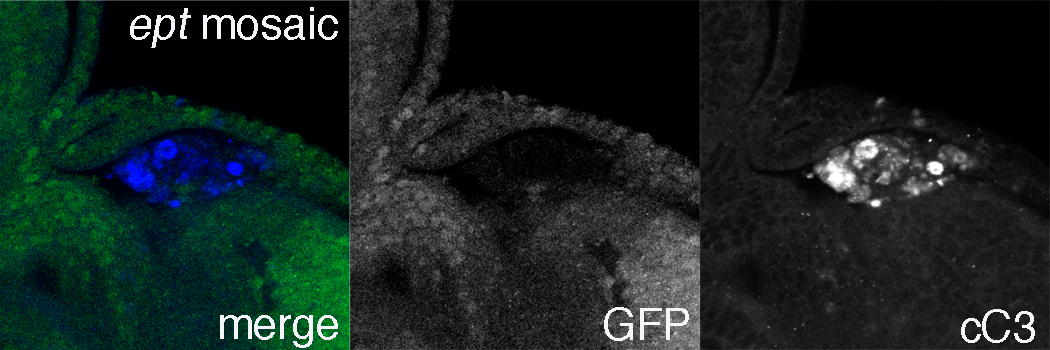

Supplement: Figure S1 — Rates of death in ept/tsg101 mutant eye-antennal clones. A clone of ept/tsg101 mutant cells in the eye disc marked by the absence of GFP (green) stain brightly for the cleaved, activated form of Caspase-3 (blue). (0.54 MB TIF) [file pone.0007083.s001.tif]
